# The Effectiveness of Prehospital Subcutaneous Continuous Lactate Monitoring in Adult Trauma: A Systematic Review

**DOI:** 10.1017/S1049023X23006623

**Published:** 2024-02

**Authors:** Jamie W. Scriven, Emir Battaloglu

**Affiliations:** 1. School of Medicine, Cardiff University, Cardiff, Wales; 2. West Midlands Central Accident, Resuscitation & Emergency Team, Birmingham, England; 3. University Hospitals Birmingham NHS Foundation Trust, Birmingham, England

**Keywords:** Emergency Medical Services, lactic acid, resuscitation, wounds and injuries

## Abstract

**Introduction:**

Existing diagnostics for polytrauma patients continue to rely on non-invasive monitoring techniques with limited sensitivity and specificity for critically unwell patients. Lactate is a known diagnostic and prognostic marker used in infection and trauma and has been associated with mortality, need for surgery, and organ dysfunction. Point-of-care (POC) testing allows for the periodic assessment of lactate levels; however, there is an associated expense and equipment burden associated with repeated sampling, with limited feasibility in prehospital care. Subcutaneous lactate monitoring has the potential to provide a dynamic assessment of physiological lactate levels and utilize these trends to guide management and response to given treatments.

**Study Objective:**

The aim of this study was to appraise the current literature on dynamic subcutaneous continuous lactate monitoring (SCLM) in adult trauma patients and its use in lactate-guided therapy in the prehospital environment.

**Methods:**

The systematic review was conducted in accordance with the PRISMA guidelines and registered with PROSPERO. Searched databases included PubMed, EMBASE via Ovid SP, Cochrane Library, and Web of Science. Databases were searched from inception to March 29, 2022. Relevant manuscripts were further scrutinized for reference citations to interrogate the fullness of the adjacent literature.

**Results:**

Searches returned 600 studies, including 551 unique manuscripts. Following title and abstract screening, 14 manuscripts met the threshold for full-text sourcing. Subsequent to the scrutiny of all 14 manuscripts, none fully met the specified eligibility criteria. Following careful examination, no article was found to cover the exact area of scientific inquiry due to disparity in technological or environmental characteristics.

**Conclusion:**

Little is known about the utility of dynamic subcutaneous lactate monitoring, and this review highlights a clear gap in current literature. Novel subcutaneous lactate monitors are in development, and the literature describing the prototype experimentation has been summarized. These studies demonstrate device accuracy, which shows a close correlation with venous lactate while providing dynamic readings without significant lag times. Their availability and cost remain barriers to implementation at present. This represents a clear target for future feasibility studies to be conducted into the clinical use of dynamic subcutaneous lactate monitoring in trauma and resuscitation.

## Introduction

Trauma is a leading cause of death and morbidity world-wide. Most deaths tend to occur within 48 hours of injury, with hemorrhage being a significant cause.^1–3^ However, major trauma is no longer only a condition of the young, as trauma in the elderly population is on the rise.^4^ More than five million people die each year due to their injuries, accounting for nine percent of deaths world-wide.^5^ In England alone, there are an estimated 20,000 major trauma cases each year, with 5,400 subsequent deaths.^6^ Early prehospital interventions are a current research focus, particularly on the use of biomarkers. One of those biomarkers is lactate.

Lactate is a known diagnostic and prognostic marker used in infection, trauma, and out-of-hospital cardiac arrest.^7–10^ It has been associated with increased mortality, surgery requirement, and organ dysfunction.^11,12^ The Advanced Trauma Life Support (ATLS) course teaches four shock classes to diagnose and manage hypovolemic shock, which utilize estimated blood loss and patient vital signs, such as blood pressure, heart rate, respiratory rate, as well as urine output and mental status.^13^ However, questions have been raised over its value in the initial diagnosis and management of shock.^14,15^ Vital signs are known to respond late to intervention and a change in clinical stability, particularly in acute hemorrhage, and may not accurately reflect the patient’s current hemodynamic status.^11,14,16,17^

Serum lactate concentration has been correlated with the presence of shock, and trend increases in prehospital lactate have been strongly associated with the need for prehospital life-saving interventions.^18–20^ Lactate clearance (through serial lactate measurement) can provide data on the adequacy of initial resuscitation in trauma and aid in prognostication.^7,21,22^ Serial lactate measurement enables monitoring of the response to administered prehospital therapy and has been evidenced to increase survival in the intensive care setting.^23–26^ The use of point-of-care (POC) testing in both the prehospital and in-hospital environment allows for this periodic assessment of lactate; however, there is an associated expense and labor requirement for repeated sampling. The introduction of subcutaneous continuous lactate monitoring (SCLM) would provide a constant and consistent opportunity to monitor and utilize the telemetry trend to guide management.

The National Institute for Health and Care Excellence (NICE; London, United Kingdom) has published research recommendation NG39/2, which aims to determine the clinical and cost-effectiveness of lactate monitoring in major trauma, and highlights the need for additional research.^27^ This systematic review aims to summarize and appraise the current literature related to the use of SCLM in trauma patients in the prehospital environment. The review findings will then focus the future research proposals in this innovative area.

### Study Question

The primary question in this study was: “Is prehospital subcutaneous continuous lactate monitoring in adult trauma patients clinically and cost-effective compared to the standard methods of guiding resuscitation?”

## Methodology

This systematic review has been devised and conducted in accordance with the Preferred Reporting Items for Systematic Reviews and Meta-Analyses (PRISMA) 2020 guidelines.^28^ The protocol for this systematic review was registered with PROSPERO (University of York; York, United Kingdom; CRD42022320900). An error was made in the initially uploaded protocol. In the uploaded document, the PubMed (National Center for Biotechnology Information, National Institutes of Health; Bethesda, Maryland USA) search strategy contained the terms “pre hospital” and “out of hospital” twice. One of each term was initially missing the hyphen. Hyphens were added and corrected before the search was run. The updated document uploaded to PROSPERO and indexed in this manuscript indicates the true search strategy.

### PICO Statement

The population investigated were adult patients (≥ 16 years old) experiencing a traumatic injury and undergoing prehospital resuscitation. The intervention of interest was dynamic SCLM, facilitating lactate-guided resuscitation. The comparator group were those undergoing alternative methods of guided resuscitation. Outcomes were based on mortality, length of hospital and critical care stay, and the quantity and type of resuscitation fluids and blood products received.

### Eligibility Criteria

All criteria were agreed upon before beginning data collection. Manuscripts detailing the use of subcutaneous lactate monitoring in the prehospital environment in adult (≥ 16 years old) trauma patients were eligible. Eligible manuscripts had to show evidence of dynamic monitoring being utilized for goal-directed therapy. A prior scoping review revealed evidence of limited studies, so all study types were included. No date requirements were set. Animal and non-human studies, manuscripts where the full text could not be sourced, and non-English language manuscripts were excluded.

### Data Sources and Search Strategy

Searched databases included PubMed, EMBASE via Ovid SP (Elsevier; Amsterdam, Netherlands), Cochrane Library (Wiley; Hoboken, New Jersey USA), and Web of Science (Clarivate Analytics; London, United Kingdom). Search terms included MeSH and tiab terms with keywords including “trauma,” “injury,” “continuous lactate,” “subcutaneous lactate,” “dynamic lactate,” “pre-hospital,” “prehospital,” “pre hospital,” “out-of-hospital,” and “out of hospital.” Table 1 demonstrates the complete search strategy for each database. All databases were searched from inception, with restrictions to include only English-language publications. The final search was carried out on March 29, 2022. The references of all relevant manuscripts were also screened to detect any other appropriate publications.

**Table 1.** Search Strategy for the Chosen Databases and Date Periods

Database	Search Strategy	Date Period
PubMed	(“wounds and injuries“[MeSH Terms] OR “trauma“[Title/Abstract] OR “injur*“[Title/Abstract]) AND (“lactic acid“[MeSH Terms] OR “continuous lact*“[Title/Abstract] OR “subcutaneous lact*“[Title/Abstract] OR “dynamic lact*“[All Fields]) AND (“emergency medical services“[MeSH Terms] OR “pre hospital*“[Title/Abstract] OR “prehospital*“[Title/Abstract] OR “pre-hospital*“[Title/Abstract] OR “out of hospital“[Title/Abstract] OR “out-of-hospital“[Title/Abstract])	From inception - March 29, 2022
EMBASE	(exp injury/ OR (trauma OR injur*).ti,ab,kf.) AND (exp lactic acid/ OR (’continuous lact*‘ OR ‘subcutaneous lact*‘ OR ‘dynamic lact*‘).ti,ab,kf.) AND (exp emergency health service/ OR (pre-hospital* OR prehospital* OR ‘pre hospital*’ OR ‘out of hospital’ OR out-of-hospital).ti,ab,kf.)	From inception - March 29, 2022
Cochrane Library	MeSH descriptor: [Wounds and Injuries] explode all trees (trauma OR injur*):ti,ab,kw MeSH descriptor: [Lactic Acid] explode all trees (“continuous lact*” OR “subcutaneous lact*” OR “dynamic lact*”):ti,ab,kw MeSH descriptor: [Emergency Medical Services] explode all trees (pre-hospital* OR prehospital* OR “pre hospital*” OR “out of hospitaOR out-of-hospital):ti,ab,kw #1 OR #2 #3 OR #4 #5 OR #6 #7 AND #8 AND #9	From inception - March 29, 2022
Web of Science	TS = (trauma OR injur*) AND TS = (“continuous lact*” OR “subcutaneous lact*” OR “dynamic lact*”) AND TS = (pre-hospital* OR prehospital* OR “pre hospital*” OR “out of hospital” OR out-of-hospital)	From inception - March 29, 2022

### Data Selection

Search results were combined using EndNote (Clarivate Analytics; Philadelphia, Pennsylvania USA) and duplicates were removed. All unique manuscripts were uploaded to the Rayyan interface,^29^ and titles and abstracts were obtained for each manuscript. One author (JS) initially screened all the titles and abstracts according to the proposed eligibility criteria and irrelevant or incomplete results were removed. The second author (EB) then independently verified the screened manuscripts.

The full text was sourced for all manuscripts consistent with the eligibility criteria or where further reading was needed before a decision could be made. Each full-text manuscript was assessed against the complete inclusion and exclusion criteria by one author (JS) and a decision was made. A second author (EB) independently verified the study selection process. The authors maintained contact throughout, and no disagreements were made following the screening process. For excluded articles, a rationale for exclusion was recorded (Table 2).

**Table 2.** Manuscripts that Underwent Full-Text Review with Rationales for Exclusion

Author	Year	Title	Reason for Exclusion
Brown JB, et al	2016	Prehospital lactate improves accuracy of prehospital criteria for designating trauma activation level	Wrong outcome Wrong intervention
Coats TJ, et al	2002	Early increases in blood lactate following injury	Wrong outcome Wrong intervention
Feliciano D, et al	2010	Discussion: Lactate is a better predictor than systolic blood pressure for determining blood requirement and mortality: could prehospital measures improve trauma triage?	Wrong outcome Wrong intervention
Fukuma H, et al	2019	Prehospital lactate improves prediction of the need for immediate interventions for hemorrhage after trauma	Wrong outcome Wrong intervention
Gaarder C, et al	2015	Prehospital point-of-care monitoring and goal-directed therapy: does it make a difference?	Wrong outcome Wrong intervention
Galvagno SM, et al	2018	Prehospital point of care testing for the prediction of lifesaving interventions in trauma patients	Wrong outcome Wrong intervention
Gonzalez-Robledo J, et al	2015	Prognostic factors associated with mortality in patients with severe trauma: from prehospital care to the intensive care unit	Wrong outcome Wrong intervention
Guyette F, et al	2011	Prehospital serum lactate as a predictor of outcomes in trauma patients: a retrospective observational study	Wrong outcome Wrong intervention
Lewis CT, et al	2016	Prehospital point-of-care lactate following trauma: a systematic review	Wrong outcome Wrong intervention
Martin-Rodriguez F, et al	2019	Prognostic value of lactate in prehospital care as a predictor of mortality and high-risk patients with trauma	Wrong outcome Wrong intervention
Martín-Rodríguez F, et al	2019	Prognostic value of lactate in prehospital care as a predictor of early mortality	Wrong outcome Wrong intervention
Martín-Rodríguez F, et al	2020	Accuracy of prehospital point-of-care lactate in early in-hospital mortality	Wrong outcome Wrong intervention
Umebachi R, et al	2018	Measurement of blood lactate, D-dimer, and activated prothrombin time improves prediction of in-hospital mortality in adults’ blunt trauma	Wrong outcome Wrong intervention Wrong population
Vandromme MJ, et al	2010	Lactate is a better predictor than systolic blood pressure for determining blood requirement and mortality: could prehospital measures improve trauma triage?	Wrong outcome Wrong intervention

### Data Extraction

The authors planned to extract all required data into a Microsoft Excel spreadsheet (Microsoft Corporation; Redmond, Washington USA). This process was to be carried out by one author, and the data then independently confirmed by the second author.

The process was intended to extract study-level data, including first author, publication date, country of origin, study design, and study size; patient-level data, including patient demographics, mechanism of injury, injury severity, method of guiding resuscitation, lactate concentration, volume and type of intravenous (IV) fluid administered, volume and type of blood products administered, and any other treatments received; and outcome measures, including survival to hospital discharge, prehospital patient mortality, in-hospital patient mortality, length of hospital stay, and length of critical care stay.

### Risk of Bias and Certainty of Evidence

The Critical Appraisal Skills Programme (CASP) Checklists^30^ were designated to assess the risk of bias for each relevant manuscript across multiple domains. The CASP checklists consist of specific criteria dependent on the research type, with a checklist available for each. One author was expected to complete a CASP checklist for each relevant manuscript, with a second author independently verifying the results. A risk of bias table was planned to summarize the findings.

### Data Synthesis

Due to the anticipated heterogenicity of the findings, the authors planned to synthesize the data and demonstrate the results through a narrative review in a tabulated format in accordance with PRISMA guidelines. To confirm statistical heterogenicity, forest plots were to be used and visually inspected, with the degree of overlap in confidence intervals and appearance of outliers then used to gauge the level of heterogeneity.

Key data extracted from the manuscripts would be summarized and displayed using evidence tables. The heterogenicity of the findings, effect of the intervention, and risk of bias would be compiled and presented using narrative and visual formats.

## Results

**Figure 1.**
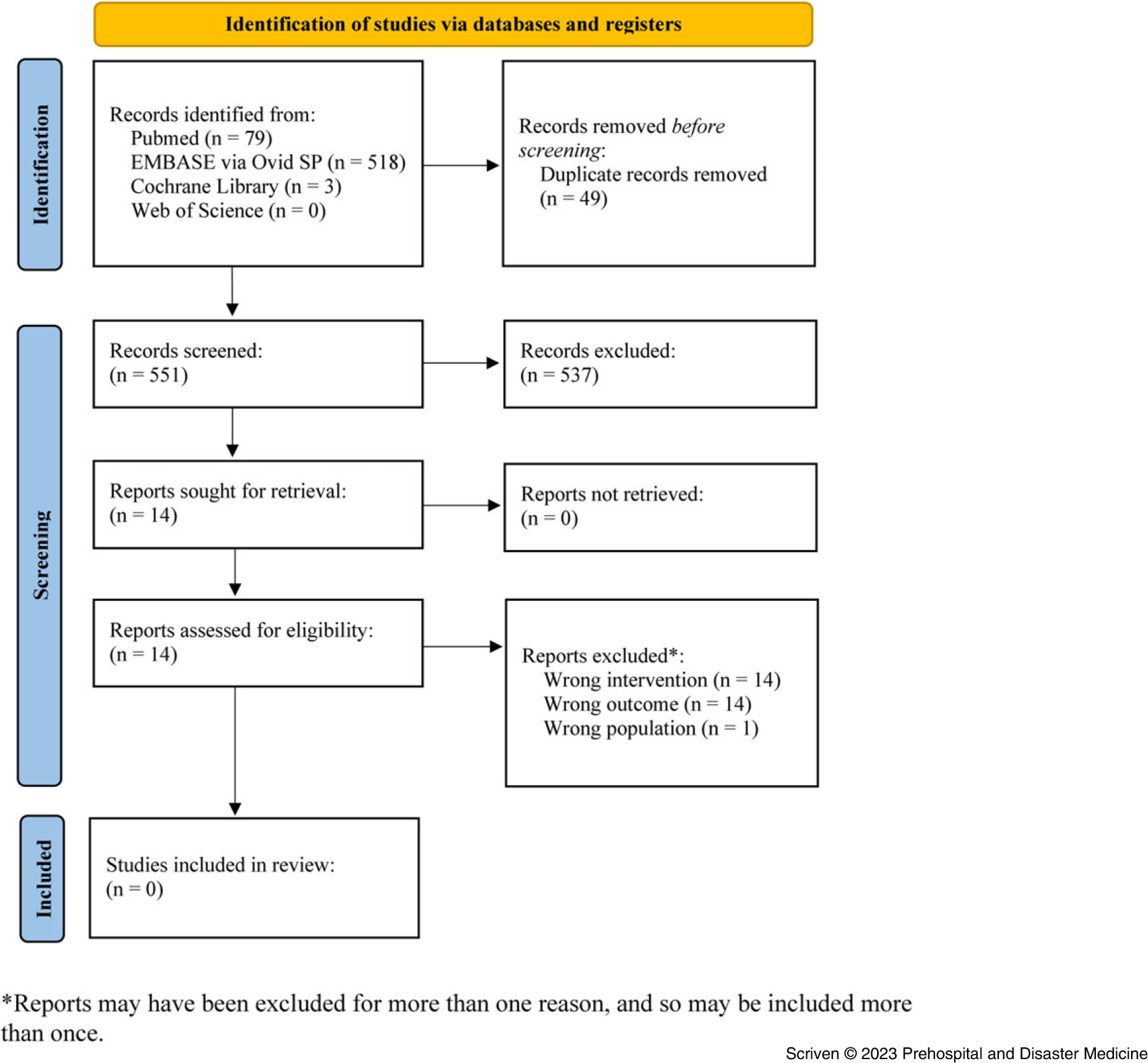
PRISMA Flowchart Demonstrating the Study Selection Process.

Six hundred manuscripts were identified across all searched databases, with 551 results after removing duplicates. Fourteen studies were identified as potentially relevant following title and abstract screening (Table 2). The full text was available for all 14 studies. After screening using the specified eligibility criteria, none of the relevant studies fully met the criteria. Figure 1 demonstrates the screening process in a PRISMA flow diagram.^28^

## Discussion

The results of this systemic review confer with the initial findings of limited published evidence on the utility of dynamic SCLM in adult trauma. Of the 14 manuscripts that underwent full-text review, most examined the use of capillary or venous lactate as a prognostic marker and to guide trauma triage. No studies evaluated the use of subcutaneous lactate monitoring, nor did they determine the clinical efficacy of dynamic lactate-guided resuscitation.

This empty review highlights a clear gap in current evidence of the efficacy and cost-effectiveness of dynamic lactate monitoring in adult trauma patients through continuous subcutaneous monitoring. Lewis, et al^21^ found in a 2016 systematic review that prehospital POC lactate monitoring showed evidence of feasibility and clinical utility, especially within the first 15 minutes of emergency services arrival. They argued that with an estimated half-life of 20 minutes, dynamic lactate monitoring may highlight a persistent state of hypoxia requiring additional resuscitative measures. Therefore, SCLM would permit the early identification of this state and expedite corrective treatment. Whilst repetitive POC lactate testing can provide a periodic assessment of lactate concentration and clearance, the lack of dynamic monitoring and the over-reliance on repeat user testing can be troublesome.

### Serial Lactate Monitoring and Lactate-Guided Resuscitation

Lactate is a proven diagnostic marker in trauma, even in those displaying no clinical features of shock.^23,31^ The variation in lactate from responder arrival at the scene to patient arrival at the emergency department has been evidenced as an independent prognostic marker, even after vital signs have been accounted for.^23^ Multiple sources have denoted the use of lactate as a clinically relevant endpoint in resuscitation in polytrauma patients and its use in guiding resuscitation.^7,23,32^ In a multicenter, open-label, randomized controlled trial based in the intensive care unit (ICU), Jansen, et al^25^ found that lactate-guided therapy decreased in-hospital mortality, inotrope use, time on mechanical ventilation, and length of ICU stay.

Recent evidence has shown that hyperlactatemia is not exclusive to anaerobic conditions but can also result from aerobic glycolysis, independent of tissue hypoxia, via beta-adrenergic stimulation. Pain and stress are two ever-present factors in prehospital trauma patients, both of which increase the stimulation of beta-adrenergic receptors through the release of adrenaline.^33,34^ This link has subsequently been demonstrated by a reduction in venous lactate in prehospital trauma patients who received IV analgesia before lactate testing.^33^ Beta-adrenergic agonists can also lead to a raised lactate and may hinder lactate-guided resuscitation in certain situations, such as traumatic cardiac arrest, where adrenaline is administered.^35^ There are several other confounding factors that may alter lactate concentration, including the presence of isolated injuries, IV fluids, hyperglycemia, as well as alcohol intake.^33,36^ Lactate clearance is predominantly mediated through direct utilization by the brain, heart, and skeletal muscle and by the conversion to glucose through the Cori cycle, which takes place in the liver.^37^ Hepatic and renal dysfunction both contribute to reduced lactate clearance.^38^ These confounding factors pose a challenge to the utility of lactate as a resuscitation endpoint prehospitally, as relatively little background is known, and the involvement of alcohol and narcotics may be undetermined.

The current literature on the use of lactate-guided resuscitation in the prehospital environment is sparse. As mentioned previously, the 2016 systematic review by Lewis, et al^21^ exploring prehospital POC lactate following trauma concluded that lactate-guided resuscitation had not been tested in the prehospital environment. The authors could find no further studies directly examining the use of prehospital lactate-guided resuscitation.

### Subcutaneous Lactate Monitoring Devices

Minimally invasive biosensor patches have demonstrated good efficacy in measuring the lactate concentration of interstitial fluid (ISF) in real time. These novel devices are yet to reach the market, however, clinical trials have revealed good patient acceptability, and close correlation with venous lactate concentration and dynamics, with a lag time of around five minutes.^39,40^ These studies demonstrated the clinical efficacy of the devices on healthy volunteers undergoing 30 minutes of moderate exercise to increase serum lactate concentration. However, a potential downfall identified is that ISF lactate clearance in the skin may be slower compared to the blood. Physiological variability, reduced skin perfusion, and differences in activity baseline may contribute to this phenomenon.^39^ This issue of decreased tissue perfusion raises an important point: trauma patients experiencing hypovolemic shock are, by definition, hypo-perfusing their tissues. Consequently, this may lead to artificially high ISF lactate levels, despite a falling serum concentration.

In contrast, a study by Kopterides, et al^41^ found the opposite. Using micro-dialysis catheters placed into the subcutaneous tissues of mechanically ventilated patients, they found tissue lactate to be better correlated to venous lactate in shocked patients compared to those who were not shocked. They also found that tissue lactate changes may precede changes in serum lactate, signaling an opportunity for subcutaneous lactate monitors to trigger earlier clinical intervention. Ultimately, these novel devices require further clinical trials in acutely unwell patients to determine their efficacy in hypoperfused states and at predicting lactate trends.

### Future Advancements

Further research is necessary on the utility of lactate-guided resuscitation in prehospital trauma patients and the use of dynamic monitoring. Ultimately, SCLM provides a safe, easy, and consistent method of lactate monitoring, with reasonable accuracy and acceptable lag times. However, the current unavailability of commercial monitors means that clinical data are somewhat limited. Additionally, the cost is a notable issue. Newly developed technology typically carries an inflated price tag, as seen with the introduction of subcutaneous glucose monitors. However, this cost is now decreasing secondary to increased manufacturing and market competition. With the development of functioning continuous lactate monitoring prototypes, an evolution in the accessibility and utility of this technology will hopefully be seen in the coming years.

## Limitations

This review found no relevant literature on the area of interest. This is presumed to be due to the lack of current evidence or data, however, manuscripts may not have been identified by the search strategy. Despite this, steps were taken to avoid missing data through a thorough search strategy and the review of all the referenced manuscripts from the 14 that underwent full-text review. With no data, a conclusion cannot be drawn for or against the clinical utility of SCLM. Nevertheless, it reveals a significant literature gap and highlights an opportunity for further research and development.

## Conclusions

Little is known about the utility of SCLM in adult trauma, and this review highlights a clear gap in current literature. Novel subcutaneous lactate monitors have shown a close correlation with venous lactate whilst providing a dynamic reading with acceptable lag times; however, their commercial availability and cost remain a potential barrier. This lack of data evidences the feasibility of further research into their use in trauma and in guiding resuscitation. Other studies have demonstrated the benefits of lactate-guided resuscitation in-hospital, but a number of confounding factors need to be considered when applying this technology to the prehospital environment.

## Supplementary material

For supplementary material accompanying this paper visit https://doi.org/10.1017/S1049023X23006623. click here to view supplementary material
